# A New Method to Scan Genomes for Introgression in a Secondary Contact Model

**DOI:** 10.1371/journal.pone.0118621

**Published:** 2015-04-14

**Authors:** Anthony J. Geneva, Christina A. Muirhead, Sarah B. Kingan, Daniel Garrigan

**Affiliations:** 1 Department of Biology, University of Rochester, Rochester, New York, United States of America; 2 Ronin Institute, Montclair, New Jersey, United States of America; University of Arkansas, UNITED STATES

## Abstract

Secondary contact between divergent populations or incipient species may result in the exchange and introgression of genomic material. We develop a simple DNA sequence measure, called *G*_min_, which is designed to identify genomic regions experiencing introgression in a secondary contact model. *G*_min_ is defined as the ratio of the minimum between-population number of nucleotide differences in a genomic window to the average number of between-population differences. Although it is conceptually simple, one advantage of *G*_min_ is that it is computationally inexpensive relative to model-based methods for detecting gene flow and it scales easily to the level of whole-genome analysis. We compare the sensitivity and specificity of *G*_min_ to those of the widely used index of population differentiation, *F*_ST_, and suggest a simple statistical test for identifying genomic outliers. Extensive computer simulations demonstrate that *G*_min_ has both greater sensitivity and specificity for detecting recent introgression than does *F*_ST_. Furthermore, we find that the sensitivity of *G*_min_ is robust with respect to both the population mutation and recombination rates. Finally, a scan of *G*_min_ across the X chromosome of *Drosophila melanogaster* identifies candidate regions of introgression between sub-Saharan African and cosmopolitan populations that were previously missed by other methods. These results show that *G*_min_ is a biologically straightforward, yet powerful, alternative to *F*_ST_, as well as to more computationally intensive model-based methods for detecting gene flow.

## Introduction

Secondary contact occurs when sympatry is restored between two or more populations that have evolved for some amount of time in allopatry. For evolutionary biologists, secondary contact between diverging populations can provide a compelling natural experiment. For example, the frequency and symmetry of hybrid matings can yield insight into the roles of sexual selection [1] and/or reinforcement [2] in speciation. Likewise, the frequency of backcrossing and subsequent introgression can reveal the extent to which postzygotic isolating mechanisms have accumulated [3]. In this context, studies of naturally occurring secondary contact offer a distinct advantage over laboratory-based studies of reproductive isolation—the patterns of introgression represent the fitness of hybrid genotypes in natural environments, replete with a variety of ecological selection pressures. Lastly, studies of secondary contact are not limited merely to satisfying the intellectual curiosity of evolutionary biologists: hybridization and introgression from closely related invasive populations can be a significant extinction threat for endangered endemic populations [4,5].

With the advent of comparative population genomics, there is now the potential to 1) quantify the frequency and tempo of introgression between natural populations experiencing secondary contact at the level of entire genomes, and 2) identify which genomic regions are exchanged. A variety of methods have been developed to estimate the rate and directionality of gene flow between diverging populations [6–9] Generally, these estimate historical population demography to assess if the observed data fit with an isolation model, and if not, estimate the direction and magnitude of gene flow necessary to explain the observed data. Comparatively fewer methods have been developed to localize introgression—identifying which genomic regions have experienced exchange—and most are tailored to have utility in particular taxa, for example requiring both “pure” and admixed samples or requiring that one population was formed by a recent dispersal event [10,11]. Many investigators rely upon unusually low observed values of the traditional fixation index, *F*_ST_ [12], to identify introgressing genomic regions (e.g., [13–15]). We suggest that *F*_ST_ may not be ideally suited for this particular application: it is derived from the variance in allele frequencies among populations and may lack power to detect introgression in cases of secondary contact [16]. This is because for *F*_ST_ to take on values close to zero following secondary contact, alleles must not only be shared across populations, but their frequencies in the two populations must also be equal. This is not necessarily expected in a secondary contact model, in which introgression is either very recent or otherwise limited. In this paper, we consider whether whole-genome sequence data can be leveraged to obtain both greater sensitivity and specificity to detect introgression than using *F*_ST_ alone.

While there are a variety of alternatives to *F*_ST_ for detecting introgression [6,8–11,17–19], our aim is to develop a method that fulfills seven criteria: 1) it has minimal prior assumptions, 2) is sensitive to recent gene flow, 3) has a low rate of false positives, 4) has a straightforward biological interpretation, 5) is applicable to a wide range of taxa, 6) can localize tracts of introgression in the genome, and 7) is fast to compute on large genomic datasets. To this end, we propose a simple haplotype-based sequence measure called *G*_min_, which is can be quickly calculated in sliding windows across whole-genome alignments. *G*_min_ is the ratio of the minimum between-population haplotype distance to the mean between-population haplotype distance, calculated in windows across the genome. We present the results of extensive computer simulations demonstrating that *G*_min_ is more sensitive to recent introgression than *F*_ST_ in a secondary contact model. We also use *G*_min_ on a previously published dataset to scan the X chromosome for introgression between sub-Saharan African and cosmopolitan populations of the commensal fruit fly *Drosophila melanogaster*.

## Materials and Methods

### Rationale for the *G*_min_ measure

Assume that we have nucleotide sequences of multiple individuals sampled from two populations, such that there are a total *n*_1_ sequences from population 1 and *n*_2_ sequences from population 2. The average number of pairwise nucleotide differences between sequences from the two populations is defined as

$${\bar{d}}_{XY}=\frac{1}{n_{1}n_{2}}\sum_{X=1}^{n_{1}}\sum_{Y=1}^{n_{2}}d_{XY},$$ 1

in which *d*_*XY*_ is the Hamming distance (or, *p*-distance) between sequence *X* from population 1 and sequence *Y* from population 2 [20]. Similarly, let min(*d*_*XY*_) be the minimum value of *d*_*XY*_ among all *n*_1_×*n*_2_ comparisons. We can then define the ratio,

$$G_{\min}=\frac{\min\left(d_{XY}\right)}{{\bar{d}}_{XY}}$$ 2

**Fig 1:**
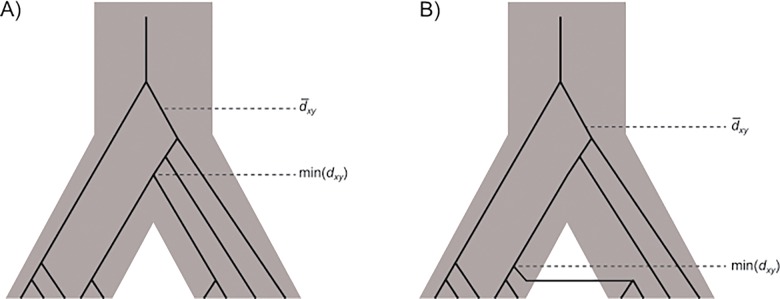
Illustration of the average and minimum between population coalescent times for models that include A) population divergence in isolation, and B) secondary contact. For sufficiently high rates of mutation, these two times are the main determinants for the observable quantities: the mean number of between population nucleotide differences, ${\bar{d}}_{XY}$ and the minimum between population differences min(*d*_*XY*_).

The ratio *G*_min_ ranges from zero to unity and has the property that if *n*_1_ = 1 and *n*_2_ = 1, then. *G*_min_ = 1. Under a strict model of isolation (i.e., no historical gene flow), a lower bound is imposed upon *G*_min_ by the divergence time between the two populations. However, for population divergence models that include recent gene flow the lower bound is determined by the timing of the most recent gene flow event (for example, see **Fig 1**). A coalescent approximation for the expectation of *G*_min_ is provided in S1 Text. We performed coalescent simulation to contrast *G*_min_ with *F*_ST_ calculated with the expression given by [21],

$$F_{ST}=1-\frac{{\bar{d}}_{XX}+{\bar{d}}_{YY}}{2{\bar{d}}_{XY}}$$ 3

### Behavior of the *G*_min_ ratio

To characterize the behavior of the *G*_min_ ratio, two sets of coalescent simulations were generated. The first set was intended only to examine the distribution of *G*_min_ under the null model of neutral population divergence with no gene flow (isolation). The second set of simulations was designed to contrast the sensitivity and specificity of *G*_min_ with those of *F*_ST_, using a binary classification procedure. This second set considers a large parameter space for a secondary contact model, which includes an ancestral population of size *N* that splits into two descendant populations at time *τ*_*D*_ (measured in units of *N* generations). We focus on cases in which each of the descendant populations also has size *N* (however, for treatment of the effects of varying population size in secondary contact models, see [22]). Subsequently, at time *τ*_*M*_ (also measured in units of *N* generations) before the present, the source population is allowed to send migrants instantaneously to the other population. Instantaneous migration was assumed, rather than specifying a time for the onset of continuous gene flow, because it more discretely captures the effect of the timing of secondary contact. The number of migrating lineages is governed by the “migration probability” parameter, *λ*. For example, at time *τ*_*M*_, let there be *k* ancestral lineages present in the source population, so that the number of lineages chosen to migrate is a binomial distributed random variable with expectation *kλ*. We assume that gene flow is unidirectional. This model is implemented in a modified version of the coalescent simulation software MS [23], called MSMOVE [24]. This modified version has the added feature of recording which simulated genealogies experienced a migration event.

Since *G*_min_ is intended to be measured in a sliding window scan of whole-genome sequence alignments, we performed simulations that approximate variably sized genomic windows. This was achieved by varying both the population mutation rate (*θ* = 4*Nμ* where *μ* is the mutation rate for a given window) and the population crossing-over rate (*ρ* = 4*Nc*, where *c* is the rate of crossing-over per window). Specifically, we used values of *θ* ∊ {10,20,50,100,150} and *ρ* ∊ {0,1,10,20,50,100,150}. To provide a more familiar frame of reference for these simulation parameters we provide the following expected values calculated as if our simulated data were derived from population sampling of DNA sequences. For a sample size of 10 individuals, it is expected that *θ* = 10 corresponds to a window size with 28 segregating sites, while *θ* = 150 approximates a window with 424 segregating sites. Similarly, *ρ* approximates the size of haplotypes within windows. For example, when *θ* = 150 and *ρ* = 0, all 424 segregating sites would be partitioned among haplotypes that span the length of the window. However, when *θ* = 150 and *ρ* = 150, there are also 424 expected recombination events, therefore each segregating site would have its own non-recombining coalescent history, on average.

For each pairwise combination of parameter values, a total of 10^4^ independent windows were simulated. This scheme assumes that large windows are being used to scan the genome for gene flow, such that genealogical histories within windows can be correlated, but that adjacent windows contain independent genealogies. Additionally, we considered two different sample size configurations. The first configuration is one in which only a single source-population sequence is available (*n*_1_ = 10 and *n*_2_ = 1) and the second sample configuration assumes that polymorphism data are available from both populations (*n*_1_ = 10 and *n*_2_ = 10). For both sample size configurations, the direction of the gene flow is from population 2 into population 1, going forward in time.

For the first set of simulations, which characterizes the behavior of *G*_min_ under the null isolation model, we considered a range of population divergence times, *τ*_*D*_ ∊ {1/25,2/25,3/25,…,8}. We performed a variance partitioning analysis to quantify the effects of the *n*_2_, *θ*, *ρ*, and *τ* parameters (as well as their interactions) on the mean and variance of both *G*_min_ and *F*_ST_. We first fit a linear model that includes all parameters and their interactions. We then quantified the variance explained by each parameter by calculating the partitioned sum of squares. For all analyses, we tested the non-independence of parameters and for any potential bias-inducing effects of model complexity by comparing variance partitioning for each parameter after 1) iterating the order of parameters in the model, 2) running models both with and without interaction terms, and 3) serially removing parameters. All post-processing and analyses of simulated data was performed using the R statistical environment [25].

### Sensitivity and specificity

To contrast the sensitivities of *G*_min_ and *F*_ST_ to gene flow under the alternative secondary contact model, we examine the proportion of simulated true migrant genealogies that are deemed outliers using a simple designation criterion. While this is not meant to be a formal statistical test of gene flow versus isolation, it is a convenient procedure for approximating the sensitivity and specificity of *G*_min_ and *F*_ST_. Using this procedure, we classify a genomic window as being “positive” for gene flow on the basis of its standardized deviation from the genome-wide mean (*Z*-score). We defined three levels of stringency for considering an individual window as positive for gene flow, *Z* < −1.645, *Z* < −2.326, and Z < −3.090. Let the set of windows with a *Z*-score less than the threshold be denoted as *Q*. Furthermore, simulated windows are classified as “true” gene flow windows if they contain a genealogy in which an ancestral lineage has switched populations. Therefore, any particular parameterization of the secondary contact model will yield the set *M* of true gene flow windows. Let *M*∩*Q* represent the set of true gene flow windows with a *Z*-score below the threshold value. The sensitivity of the test (*φ*) can therefore be defined as the proportion

$$\varphi =\frac{\left|M\cap Q\right|}{\left|M\right|}$$ 4

Thus, *φ* = 1, when all true gene flow windows have an outlying *Z*-score. Conversely, we define specificity (*ψ*) as

$$\psi =\frac{\left|M\cap Q\right|}{\left|Q\right|},$$ 5

such that if *ψ* = 1, then all windows with an outlying *Z*-score are true gene flow windows. For the analysis of sensitivity and specificity, the simulated parameter combinations were the same as those used in the first set of simulations described in the previous subsection. The only exceptions were that we simulated a narrower range of divergence times *τ*_*D*_ ∊ {1/100,2/100,3/100,…,1} and added two additional parameters: the relative time of gene flow, which had the range *τ*_*M*_ ∊ {*τ*_*D*_/100,2*τ*_*D*_/100,3*τ*_*D*_/100,…,*τ*_*D*_} (for *τ*_*D*_ > 0) and migration probability in the set, *λ* ∊ {0.001,0.005,0.01,0.05,0.1}. In addition to assessing the sensitivity and specificity of *G*_min_ and *F*_ST_, we also evaluated the effect of each varied parameter on sensitivity and specificity. Variance partitioning was performed as described in the previous subsection.

### Application to *Drosophila melanogaster* data

We developed *G*_min_ in anticipation of high-quality short-read assemblies of population-level samples from more than one population. Such data have just begun to emerge from a variety of organisms. To contrast the sensitivity of *G*_min_ with that of *F*_ST_, we apply it to a subset of the highest quality available resequence dataset: X chromosome polymorphism of two populations of *Drosophila melanogaster* [10]. The two populations include a cosmopolitan population from France and a sub-Saharan African population from Rwanda. While these two populations generally show low levels of sequence divergence (chromosome average *F*_*ST*_ = 0.183 and ${\bar{d}}_{XY}=0.0085$), a recent study was able to detect a signal of recent cosmopolitan admixture in several African populations, including the deeply sampled Rwandan population [10].

We obtained 76 bp paired-end Illumina reads from seven French and nine Rwandan lines from the NCBI short read archive (see **S1 Table** for details on the sampled lines). All reads were aligned to the reference genome of *D*. *melanogaster*, build 5.45 (http://flybase.org), using the BWA software, version 0.6.2 [26]. The resulting alignments for individual lines in the BAM format were merged using the SAMTOOLS software package [27]. The values of *F*_ST_ and *G*_min_ were calculated in non-overlapping 50 kb windows using the POPBAM software package [28]. We only analyzed nucleotide sites that met the following criteria: read depth per line greater than 5, Phred-scaled scores for the minimum root-mean squared mapping quality greater than or equal to 25, and a SNP quality that is at least 25; we also only incorporated reads with a minimum mapping quality of 20 and an individual base quality of at least 13. Of the 443 X chromosome 50 kb windows, seven (1.58%) had less than 25% of the reference genome positions passing the above filters and were subsequently ignored. Lastly, we construct neighbor-joining trees based on uncorrected Hamming distance in 50 kb windows using POPBAM. For the sake of consistency, individual windows were identified as outliers if *Z* < −1.645. We compare our analysis to that of Pool et al. [10], who utilized a Hidden Markov Model method based on the pairwise distances between sub-Saharan African and cosmopolitan genomes. In windows of 1000 non-singleton SNPs, each Rwandan line was assigned a posterior probability of admixture. We identified previously known admixed regions as those whose sum of posterior probabilities across lines is greater than 0.50 (see S5 Table from Pool *et al*. 2012).

## Results

### Behavior of the *G*_min_ ratio under an isolation model

**Fig 2:**
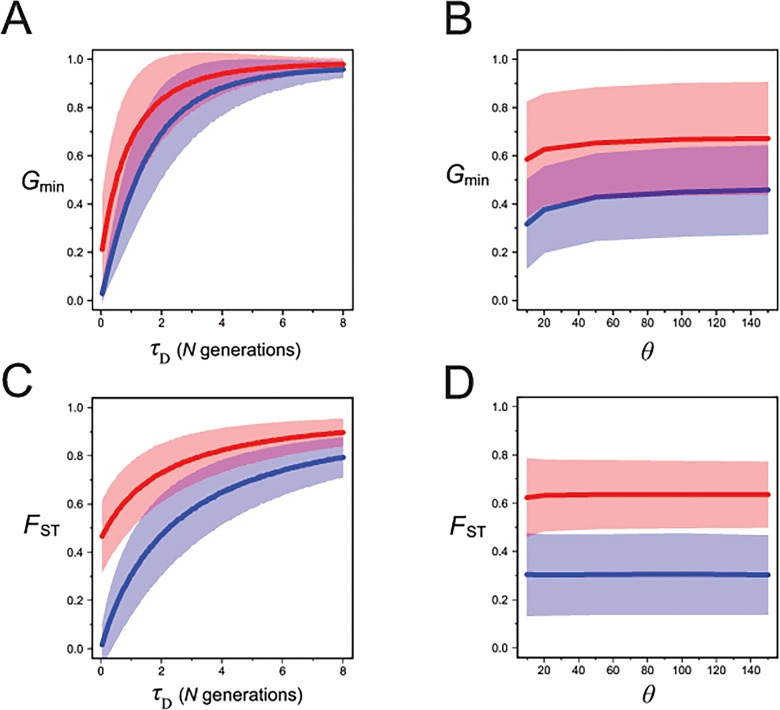
Expected values of *G*_min_ in a pure isolation model. A) The mean simulated values of *G*_min_ plotted against divergence time for a model of divergence in isolation. B) Mean simulated values of *G*_min_ plotted against population mutation rate under an isolation model with divergence occurring at time *τ*_*D*_ = *N* generations ago. Also shown is the mean simulated values of *F*_ST_ plotted against C) divergence time and D) population mutation rate under an isolation model. The shaded areas delimit the mean ± one standard deviation. The blue lines represent sample sizes in the two populations of *n*_1_ = 10 and *n*_2_ = 10, while the red lines represent sample sizes of *n*_1_ = 10 and *n*_2_ = 1. The simulations shown here do not include the effects of intra-locus recombination.

*G*_min_ is the ratio of min(*d*_*XY*_), the minimum number of nucleotide differences between haplotypes sampled from different populations, to ${\bar{d}}_{XY}$ the average number of between-population differences (Eq. 2). In a strict isolation model of divergence, we expect that both min(*d*_*XY*_) and ${\bar{d}}_{XY}$ will increase as a function of the population divergence time, *τ*_*D*_. Ultimately, *G*_min_ is expected to approach unity for very ancient divergence times (*τ*_*D*_ >> 4*N*), because there is a high probability of only a single ancestral lineage remaining in each population. Conversely, for very recent divergence times, *G*_min_ is expected to be much less than unity, since it is unlikely that all coalescent events will occur only between ancestral lineages from the same population before a single coalescent occurs between lineages from different populations. Computer simulations show that both *G*_min_ and *F*_ST_ increase asymptotically to unity as the divergence time increases, but also that *G*_min_ increases at a faster rate and plateaus at an earlier divergence time (**Fig 2**).

In the isolation model, the variance of *G*_min_ is most strongly affected by the time of population divergence, *τ*_*D*_. Variation in *τ*_*D*_ alone explains approximately half of the simulated variance for both *G*_min_ and *F*_ST_ (**Table 1**). When the population mutation rate *θ* ≤ 10, *G*_min_ becomes downwardly biased (**Fig 2B**). We suspect that this bias arises for low mutation rates because, when few mutations occur on a set of correlated genealogies, *G*_min_ does not always capture the minimum time of the between-population coalescent events, rather it may reflect a randomly chosen between-population coalescent event that, by chance, has fewer mutations separating them than the true minimum event. Finally, whether a single source-population sequence is available (*n*_2_ = 1) or polymorphism data are available (*n*_2_ = 10) has a minor, but predictable, effect: *G*_min_ is always closer to unity when *n*_2_ = 1 than when *n*_2_ = 10 (**Fig 2**). It should be noted that although we report on the results for *F*_ST_ in the case of *n*_2_ = 1, this is obviously not a situation in which *F*_ST_ (as a measure of difference in allele frequencies) would be applicable. Finally, we found no evidence of bias in any of the variance partitioning analyses, so that the full models with all parameters and interaction terms have been included.

**Table 1:** Variance partitioning for *G*_min_ and *F*_ST_ under the isolation model of divergence.

Parameter	*G*_min_	SD(*G*_min_)	*F*_ST_	SD(*F*_ST_)
*τ*_*D*_	48.5	44.9	54.7	27.3
*θ*	3.9	2.4	0	1.4
*n*_2_	7.6	0.1	29.3	5.5
*ρ*	2.5	16	0	39.1
*τ*_*D*_ × *n*_2_	2.9	1.3	5.4	0.2
*τ*_D_ × *ρ*	3.9	5.3	0	2.1
Coalescent processes	29.8	28.8	10.5	23.2

Column values are the percent variance in each of the two statistics and their respective standard deviations (SD) described by each model parameter (or interaction of parameters), including the population divergence time *τ*_*D*_, the population mutation (*θ*) and recombination (*ρ*) rates, and the sample size from a second population (*n*_2_). The table only includes parameters with an effect greater than 1%.

### Sensitivity and specificity

**Fig 3:**
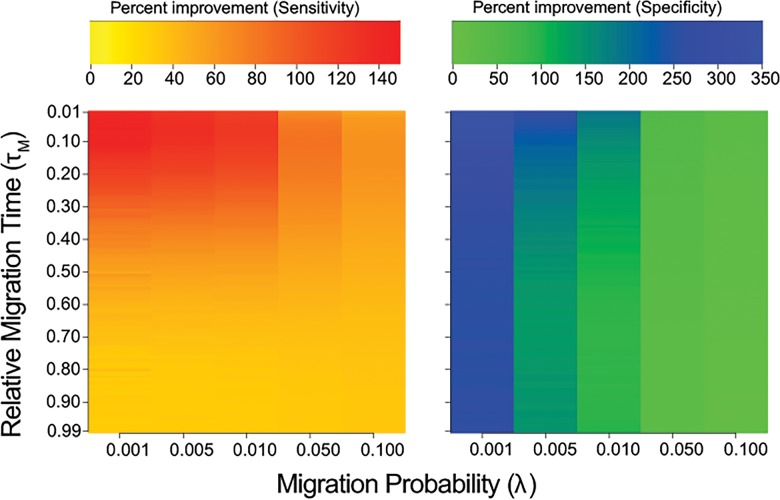
Comparison of *G*_min_ and *F*_ST_. Heatmaps of percent improvement of *G*_min_ over *F*_ST_ for sensitivity (left) and specificity (right). Improvement was calculated for varying rates of migration (migration probability) and time of migration (relative to time of population divergence) and averaged over all other parameters.

When we consider a secondary contact model, the two parameters that exert the strongest influence on the behavior of both *G*_min_ and *F*_ST_ are the time of migration relative to divergence (*τ*_*M*_) and the magnitude (*λ*) of the migration event (**S2** and **S3 Tables**). Our simulations show that *G*_min_ has increased sensitivity and specificity compared to *F*_ST_ for all combinations of the *τ*_*M*_ and *λ* parameters, regardless of the values of nuisance parameters, such as *θ* and *ρ* (**Fig 3**). The sensitivity of *G*_min_ is greatest when *τ*_*M*_ is recent and *λ* is small (**S1 Fig**). It is interesting to note that the sensitivity of *G*_min_ decreases with increasing *λ* because large amounts of migration tends to reduce the average between-population sequence distance, thereby also reducing the expected *G*_min_ and increasing its variance (**S4 Table**). However, for *F*_ST_, *λ* does not have a profound effect on its sensitivity (**S4 Table**). In contrast, increased *λ* results in a greater specificity for *G*_min_ (**Fig 3**). This means that although high *λ* results in a lower proportion of the migrant genealogies appearing in the negative *Z*-score tail, a greater proportion of all genealogies in the tail are true migrant genealogies.

Surprisingly, the rate of recombination has only a mild effect on the sensitivity of *G*_min_ and *F*_ST_ (**S2 Fig**). This may be due to the relatively intermediate levels of recombination used in the computer simulations, since the recombination rate must be very high (*ρ* > 50) to break up introgressed haplotypes when *τ*_*M*_ is very recent. This is also true of specificity (**S3 Fig**). Likewise, increasing the population mutation rate also slightly increases both the sensitivity (**S4 Fig**) and the specificity (**S5 Fig**). These results suggest that sensitivity and specificity of *G*_min_ are optimal when large genomic windows (*θ* > 10) with relatively low levels of recombination (*ρ* < 20) are considered.

A trade-off between sensitivity and specificity occurs when we contrast results from simulations of divergence from a single source population sequence (*n*_2_ = 1) with those from polymorphism data from both populations (*n*_2_ = 10). *G*_min_ has increased sensitivity when *n*_2_ = 1 compared to when *n*_2_ = 10 (**S6 Fig**). In contrast, the specificity of *G*_min_ is substantially greater when *n*_2_ = 10 (**S7 Fig**). Therefore, situations in which only a single source-population sequence is available results in *G*_min_ having increased power to detect migrant genealogies at any given locus in the genome, while polymorphism data from two populations yields increased power to detect gene flow across the genome. The specificity result is intuitive from a biological standpoint: if low levels of gene flow occur, then having more sequences per population will increase the probability of recovering an introgressed haplotype. Sensitivity increases when *n*_2_ = 1 because there is less variance in the coalescent process in the ancestral population for genealogies that do not experience gene flow and the expected *G*_min_ in an isolation model is closer to unity; this results in a higher proportion of migrant genealogies significantly departing from a genome-wide distribution.

### Application to cosmopolitan admixture in *Drosophila melanogaster*

**Fig 4:**
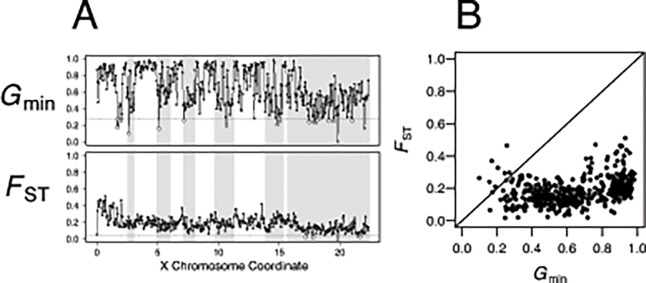
Cosmopolitan admixture in sub-Saharan African *Drosophila melanogaster*. A) *G*_min_ (above) and *F*_ST_ (below) in 50 kb windows across the X chromosome in a sample of seven French and nine Rwandan lines. Shaded regions indicate where Pool et al. [10] previously detected admixture. Open circles mark windows that are identified as outliers from the chromosome-wide distribution. B) Scatterplot of *F*_ST_ versus *G*_min_ across the X chromosome in 50 kb windows. The diagonal line is added for reference only.

We compare the ability of *G*_min_ versus *F*_ST_ to detect cosmopolitan admixture in a Rwandan population of *D*. *melanogaster*. We used POPBAM to calculate the two statistics in 436 non-overlapping 50 kb windows on the X chromosome in a sample of seven French and nine Rwandan lines (**Fig 4A**). The mean and standard error for *G*_min_ is 0.6500 ± 0.0311 and for *F*_ST_ is 0.1725 ± 0.0083. Interestingly, the range of *G*_min_ (0.0982—0.9833) is more than twice as large as that of *F*_ST_ (0.0170–0.5107) (**Fig 4B**). This expanded range of *G*_min_ is consistent with a greater sensitivity of *G*_min_, even for relatively low levels of population divergence. The outliers from the chromosome-wide *G*_min_ distribution identified cosmopolitan admixture in all of the previously identified admixture windows (**Fig 4A**). In contrast, outlier values of *F*_ST_ appear in only one of the six previously identified tracts (**Fig 4A**). The outliers of *G*_min_ also reveal two additional candidate introgression tracts on the X chromosome—a region consisting of five significant windows between coordinates 1.65–2.05 Mb, and a single window located at 12.95–13 Mb just above our arbitrary cut-off (*Z* = −1.6352); neither region was previously identified by Pool et al. [10]. The first region near the 2 Mb coordinate harbors a low frequency introgressed haplotype carried by Rwandan line, RG35. Neighbor-joining trees indicate that the RG35 sample is nested within the French samples, although the particular French line(s) with which it clusters varies across windows (**S8 Fig**). The second marginally significant window involves a similar scenario where RG35 is nested within the clade of French lines, sister to the French line FR229 (**S9 Fig**). These inferred low frequency introgressions went undetected in both our *F*_ST_ scan and the Hidden Markov Model analysis performed by Pool et al. [10] The window size used by Pool et al. [10] was based on the number of SNPs, rather than physical distance, such that windows in this sub-telomeric region are larger than 100 kb, on average. Therefore, it is possible that the large windows analyzed by Pool et al. [10] contain conflicting genealogical histories, resulting in the distance between RG35 and any particular French line not being reduced, on average.

## Discussion

Comparative population genomic datasets, or whole genome alignments of many individuals from multiple populations within a species or between closely related species, are finally becoming realized in evolutionary genetics. One of the many potential uses of these new data is to estimate the degree to which introgression occurs between populations coming into secondary contact. Also of interest is pinpointing the genomic location of introgression and characterizing the functional properties of introgressing coding material, if any. Many of the first studies to make use of whole-genome datasets rely on the traditional fixation index, *F*_ST_, to identify introgressed genomic regions. However, we have shown that *F*_ST_ has a number of inherent weaknesses for detecting introgression in a secondary contact model.

Our analyses focus on phased haplotype data, which can be especially useful for inferring details of historical population demography and gene flow [18,29,30] and haplotype sharing among populations is often used as a criterion for detecting introgression [19,31,32]. We show that haplotype-based measures of within- and between-population sequence differences, such as *G*_min_, offer better sensitivity and specificity over allele frequency measures such as *F*_ST_. Furthermore, our simulations show that *G*_min_ is robust to local variation in mutation rate and, to a lesser extent, recombination rate. The robustness of *G*_min_ to the local recombination rate primarily occurs when gene flow is both recent and limited, in which case there is a limited opportunity for recombination to break up introgressed haplotypes (**S2** and **S3 Figs**). This result suggests that choice of window size offers an avenue for distinguishing recent versus older introgression events (**S10 Fig**). Larger windows with more mutation and recombination events offer greater power to identify very recent introgression events, whereas smaller windows can identify older introgression events, albeit with less specificity than larger windows. In practice, the most useful window size will vary by the particular taxa of interest. Due to the relative ease in calculating *G*_min_, optimal window size can be rapidly evaluated over a range of genomic intervals.

Like *F*_ST_ or ${\bar{d}}_{XY},$ *G*_min_ is not a formal test statistic, rather it is a sequence measure designed to identify a distinctly bimodal pattern of between-population coalescence that is expected under models of secondary contact, but not expected in models of strict population isolation. We were unable to derive a closed-form expression for the variance of the *G*_min_ ratio in a pure isolation model, due in part to the fact that we observe a non-zero positive covariance between the numerator, min(*d*_*XY*_), and the denominator, ${\bar{d}}_{XY}$ (data not shown). Therefore, using *G*_min_ as the basis for a simple single-locus test is not currently feasible. However, like *F*_ST_, *G*_min_ can be readily incorporated into other inferential frameworks, such as approximate likelihood methods [33]. Our approach differs from more formal inferential frameworks, such as those used by the IM program [9], in that IM tests the hypothesis of whether or not gene flow has occurred; the goal of *G*_min_ is less formal, seeking instead to localize introgression genealogies in otherwise diverging genomes. In practice, a *G*_min_ scan may be an extremely useful first step for identifying candidate regions for introgression. Unlike many likelihood-based methods for detecting gene flow in a population divergence model, *G*_min_ can be quickly applied to large whole-genome datasets and interpretation of *G*_min_ requires a minimal set of assumptions. The fundamental assumption is that the individuals in the analysis came from either one population or a different population. This is in contrast to some methods for detecting admixed regions of the genome, which rely on investigators being able to assign individuals to two pure parental populations, as well as a third population of hybrid individuals [11]. Of course, knowing the hybrid status of individuals, or having more detailed information of sample geographical distribution, may enable more advanced analysis [6,17].

While *G*_min_ is more sensitive to recent gene flow than *F*_ST_, it has additional desirable properties that distinguish it from other recently proposed haplotype-based methods. For example, Harris and Nielsen [8] describe a method for detecting recent gene flow by measuring the genomic length distribution of tracts of identity-by-state. The computer simulations presented by Harris and Nielsen [8] demonstrate that their method can accurately infer the timing and magnitude of admixture events, as well as other demographic parameters, over a range of time scales. However, the identity-by-state method of Harris and Nielsen [8] may also be sensitive to 1) low quality reads and sequencing error, 2) reductions in effective population size due to background selection, and 3) accuracy of the required modeling of historical population bottlenecks. In contrast, we argue that *G*_min_ is not as sensitive to errors in sequencing or assembly, because *G*_min_ does not explicitly depend upon uninterrupted runs of shared polymorphic sites. Additionally, the lower tail of *G*_min_ is not expected to be strongly affected by background selection under a secondary contact model. This is because background selection does not affect the tempo of neutral divergence [34] and can skew within-population polymorphism towards an excess of rare alleles [35], neither of which affects *G*_min_ (however, for the effect of reductions in the effective population size, see below).

Besides recent introgression, the primary factor affecting *G*_min_ is the number of ancestral lineages present at the time of the initial population split. As a result, the distribution of *G*_min_ will be affected by any force that alters the probability density of within-population coalescent events, including changes in the effective population size or natural selection. If natural selection acts to reduce diversity in one population exclusively or, if the effective population size of one population is smaller than that of the other, we expect there to be fewer ancestral lineages present at the time of the initial population divergence. To consider the performance of *G*_min_ in these cases, we can extrapolate from our computer simulation results of different sampling schemes, in particular when *n*_1_ = 10 and *n*_2_ = 1. We find that when only a single source-population genome is used, *G*_min_ has greater sensitivity (**S6 Fig**), but reduced specificity compared to when *n*_2_ = 10 (**S7 Fig**). This suggests that forces acting to increase the rate of coalescence within populations, such as population bottlenecks, will result in increased confidence that small values of *G*_min_ can be attributed to recent gene flow, but also a diminished ability to recover all of the introgressed regions in a genome. Similarly, the reduced specificity of *F*_ST_ when there is a reduction in within-population variation is well-known [21,36,37], however *G*_min_ does not appear to be as strongly affected as *F*_ST_ (**S7 Fig**).

In conclusion, we do not wish to argue that *G*_min_ is in any way a panacea for the longstanding problem of distinguishing models of gene flow from those of pure isolation [38]. Indeed, *G*_min_ lacks sensitivity when gene flow occurred more than halfway back to the time of the population divergence or when there is a large amount of gene flow (**S1 Fig**). For example, if a genomic region is sweeping across species boundaries [39], *G*_min_ is not expected to be as informative as *F*_ST_. Therefore, it is also important to caution that genomic intervals with low *G*_min_ should be subsequently vetted to ensure that the region does not have unusually low absolute values of ${\bar{d}}_{XY}$. However, in cases of recent secondary contact, and when the rates of gene flow are not extremely high, we have shown that *G*_min_ performs well and is more reliable than *F*_ST_ (**Fig 3**). In addition, we illustrate how a simple statistical procedure employing *G*_min_ to scan the X chromosome of recently diverged cosmopolitan and sub-Saharan African populations of *Drosophila melanogaster* performs as well as more sophisticated methods (**Fig 4**). However, unlike many more sophisticated methods, the calculation of *G*_min_ is fast and broadly applicable to any taxa for which haploid genome sequences are available. *G*_min_ can be easily calculated from population genomic data using the software package POPBAM [28]. We anticipate that with the continued emergence of new haplotype sequencing methods [40,41], these types of data will be increasingly used for evolutionary studies. In this case, *G*_min_ can be an effective and biologically straightforward addition to the suite of tools available to evolutionary biologists.

## Supporting Information

S1 Fig A) Sensitivity of the *F*_ST_ and *G*_min_ measures for varying rates of migration (migration probability) and time of migration (relative to time of population divergence). The left column shows plots of sensitivity for *F*_ST_ and the right column shows sensitivity for *G*_min_. The top row shows sensitivity when outliers are defined by *Z* < −1.645, the middle row shows the same for *Z* < −2.326, and the bottom row shows sensitivity when Z < −3.090. B) Specificity of the *F*_ST_ and *G*_min_ measures for varying rates and times of migration. Layout of the plots are the same as in panel A.
(EPS)

S2 Fig Sensitivity of F_ST_ (left column) and G_min_ (right column) for varying levels of population recombination rate: *ρ* = 0 (top), *ρ* = 50 (middle), and *ρ* = 150 (bottom). (EPS)

S3 Fig Specificity of F_ST_ (left column) and G_min_ (right column) for varying levels of population recombination rate: *ρ* = 0 (top), *ρ* = 50 (middle), and *ρ* = 150 (bottom). (EPS)

S4 Fig Sensitivity of F_ST_ (left column) and G_min_ (right column) for varying levels of population mutation rate: *θ* = 10 (top), *θ* = 50 (middle), and *θ* = 150 (bottom). (EPS)

S5 Fig Specificity of F_ST_ (left column) and G_min_ (right column) for varying levels of population mutation rate: *θ* = 10 (top), *θ* = 50 (middle), and *θ* = 150 (bottom). (EPS)

S6 Fig Sensitivity of F_ST_ (left column) and G_min_ (right column) for varying sample size: *n*_2_ = 10 (top) and *n*_2_ = 1 (bottom). (EPS)

S7 Fig Specificity of F_ST_ (left column) and G_min_ (right column) for varying sample sizes: *n*_2_ = 10 (top) and *n*_2_ = 1 (bottom). (EPS)

S8 Fig Neighbor-joining trees showing the first newly identified region of gene flow on the *Drosophila melanogaster* X chromosome between coordinates 1.65–2.05 Mb. (EPS)

S9 Fig Neighbor-joining trees showing the second newly identified region of gene flow on the *Drosophila melanogaster* X chromosome between coordinates 12.95–13 Mb. (EPS)

S10 Fig G_min_ and F_ST_ scans of the *Drosophila melanogaster* X chromosome in differently sized windows. (EPS)

S1 Table The sampled lines from two populations of *Drosophila melanogaster*. (DOCX)

S2 Table Analysis of variance of sensitivity of G_min_ and F_ST_. (DOCX)

S3 Table Analysis of variance of specificity of G_min_ and F_ST_. (DOCX)

S4 Table Influence of migration probability (*λ*) on the sensitivity, specificity and variance of G_min_ and F_ST_. (DOCX)

S1 Text A new method to scan genomes for introgression in a secondary contact model. (DOCX)

## References

[1] Ritchie MG. Sexual selection and speciation. Annu Rev Ecol Syst. 2007;38: 79–102.

[2] Yukilevich R. Asymmetrical patterns of speciation uniquely support reinforcement in *Drosophila* . Evolution. 2012;66: 1430–1446. 10.1111/j.1558-5646.2011.01534.x 22519782

[3] Gompert Z, Parchman TL, Buerkle CA. Genomics of isolation in hybrids. Phil Trans R Soc B. 2012;367: 439–450. 10.1098/rstb.2011.0196 22201173 PMC3233709

[4] Rhymer JM, Simberloff D. Extinction by hybridization and introgression. Annu Rev Ecol Syst. 1996;27: 83–109.

[5] Seehausen OLE, Takimoto G, Roy D, Jokela J. Speciation reversal and biodiversity dynamics with hybridization in changing environments. Mol Ecol. 2008;17: 30–44. 18034800 10.1111/j.1365-294X.2007.03529.x

[6] Barton NH, Etheridge AM, Kelleher J, Véber A. Inference in two dimensions: allele frequencies versus lengths of shared sequence blocks. Theor Popul Biol. 2013;87: 105–119. 10.1016/j.tpb.2013.03.001 23506734

[7] Durand EY, Patterson N, Reich D, Slatkin M. Testing for ancient admixture between closely related populations. Mol Biol Evol. 2011;28: 2239–2252. 10.1093/molbev/msr048 21325092 PMC3144383

[8] Harris K, Nielsen R. Inferring demographic history from a spectrum of shared haplotype lengths. PLoS Genet. 2013;9: e1003521 10.1371/journal.pgen.1003521 23754952 PMC3675002

[9] Sousa V, Hey J. Understanding the origin of species with genome-scale data: modelling gene flow. Nat Rev Genet. 2013;14: 404–414. 10.1038/nrg3446 23657479 PMC5568773

[10] Pool J, Corbett-Detig R, Sugino R, Stevens K, Cardeno C, et al Population genomics of sub-Saharan *Drosophila melanogaster*: African diversity and non-African admixture. PLoS Genet. 2012;8: e1003080 10.1371/journal.pgen.1003080 23284287 PMC3527209

[11] Price AL, Tandon A, Patterson N, Barnes KC, Rafaels N, et al Sensitive detection of chromosomal segments of distinct ancestry in admixed populations. PLoS Genet. 2009;5: e1000519 10.1371/journal.pgen.1000519 19543370 PMC2689842

[12] Wright S. The genetical structure of populations. Ann Eugen. 1951;15: 323–354. 24540312 10.1111/j.1469-1809.1949.tb02451.x

[13] Nadachowska-Brzyska K, Burri R, Olason PI, Kawakami T, Smeds L, et al Demographic divergence history of pied flycatcher and collared flycatcher inferred from whole-genome re-sequencing data. PLoS Genet. 2013;9: e1003942 10.1371/journal.pgen.1003942 24244198 PMC3820794

[14] Neafsey DE, Barker BM, Sharpton TJ, Stajich JE, Park DJ, et al Population genomic sequencing of Coccidioides fungi reveals recent hybridization and transposon control. Genome Res. 2010;20: 938–946. 10.1101/gr.103911.109 20516208 PMC2892095

[15] Smith J, Kronforst MR. Do *Heliconius* butterfly species exchange mimicry alleles? Biol Lett. 2013;9: 20130503 10.1098/rsbl.2013.0503 23864282 PMC3730661

[16] Murray MC, Hare MP. A genomic scan for divergent selection in a secondary contact zone between Atlantic and Gulf of Mexico oysters, *Crassostrea virginica* . Mol Ecol. 2006;15: 4229–4242. 17054515 10.1111/j.1365-294X.2006.03060.x

[17] Gompert Z, Buerkle CA. Bayesian estimation of genomic clines. Mol Ecol. 2011;20: 2111–2127. 10.1111/j.1365-294X.2011.05074.x 21453352

[18] Machado CA, Kliman RM, Markert JA, Hey J. Inferring the history of speciation from multilocus DNA sequence data: the case of *Drosophila pseudoobscura* and close relatives. Mol Biol Evol. 2002;19: 472–488. 11919289 10.1093/oxfordjournals.molbev.a004103

[19] Ralph P, Coop G. The geography of recent genetic ancestry across europe. PLoS Biol. 2013;11: e1001555 10.1371/journal.pbio.1001555 23667324 PMC3646727

[20] Nei M, Li W-H. Mathematical model for studying genetic variation in terms of restriction endonucleases. Proc Natl Acad Sci USA. 1979;76: 5269–5273. 291943 10.1073/pnas.76.10.5269 PMC413122

[21] Charlesworth B. Measures of divergence between populations and the effect of forces that reduce variability. Mol Biol Evol. 1998;15: 538–543. 9580982 10.1093/oxfordjournals.molbev.a025953

[22] Geneva A, Garrigan D. Population genomics of secondary contact. Genes. 2010;1: 124–142. 10.3390/genes1010124 24710014 PMC3960861

[23] Hudson RR. Generating samples under a Wright-Fisher neutral model of genetic variation. Bioinformatics. 2002;18: 337–338. 11847089 10.1093/bioinformatics/18.2.337

[24] Garrigan D, Geneva AJ. msmove: A modified version of Hudson's coalescent simulator ms allowing for finer control and tracking of migrant genealogies. 2014; 10.6084/m9.figshare.1060474

[25] R Core Team. R: A Language and Environment for Statistical Computing. Vienna, Austria: R Foundation for Statistical Computing; 2013 10.3758/s13428-013-0330-5

[26] Li H, Durbin R. Fast and accurate short read alignment with Burrows-Wheeler transform. Bioinformatics. 2009;25: 1754–1760. 10.1093/bioinformatics/btp324 19451168 PMC2705234

[27] Li H, Handsaker B, Wysoker A, Fennell T, Ruan J, et al The Sequence Alignment/Map format and SAMtools. Bioinformatics. 2009;25: 2078–2079. 10.1093/bioinformatics/btp352 19505943 PMC2723002

[28] Garrigan D. POPBAM: tools for evolutionary analysis of short read sequence alignments. Evol Bioinform. 2013;9: 343–353. 10.4137/EBO.S12751 24027417 PMC3767577

[29] Pool JE, Hellmann I, Jensen JD, Nielsen R. Population genetic inference from genomic sequence variation. Genome Res. 2010;20: 291–300. 10.1101/gr.079509.108 20067940 PMC2840988

[30] Pool JE, Nielsen R. Inference of historical changes in migration rate from the lengths of migrant tracts. Genetics. 2009;181: 711–719. 10.1534/genetics.108.098095 19087958 PMC2644959

[31] Hufford MB, Lubinksy P, Pyhajarvi T, Devengenzo MT, Ellstrand NC, et al The genomic signature of crop-wild introgression in maize. PLoS Genet. 2013;9: e1003477 10.1371/journal.pgen.1003477 23671421 PMC3649989

[32] Kijas JW, Lenstra JA, Hayes B, Boitard S, Porto Neto LR, et al Genome-wide analysis of the world's sheep breeds reveals high levels of historic mixture and strong recent selection. PLoS Biol. 2012;10: e1001258 10.1371/journal.pbio.1001258 22346734 PMC3274507

[33] Beaumont M, Zhang W, Balding D. Approximate Bayesian computation in population genetics. Genetics. 2002;162: 2025–2035. 12524368 10.1093/genetics/162.4.2025 PMC1462356

[34] Birky CW, Walsh JB. Effects of linkage on rates of molecular evolution. Proc Natl Acad Sci USA. 1988;85: 6414–6418. 3413105 10.1073/pnas.85.17.6414 PMC281982

[35] Charlesworth D, Charlesworth B, Morgan MT. The pattern of neutral molecular variation under the background selection model. Genetics. 1995;141: 1619–1632. 8601499 10.1093/genetics/141.4.1619 PMC1206892

[36] Cruickshank TE, Hahn MW. Reanalysis suggests that genomic islands of speciation are due to reduced diversity, not reduced gene flow. Molecular Ecology. 2014;23: 3133–3157. 10.1111/mec.12796 24845075

[37] Nei M. Analysis of gene diversity in subdivided populations. Proc Natl Acad Sci USA. 1973;70: 3321–3323. 4519626 10.1073/pnas.70.12.3321 PMC427228

[38] Takahata N, Slatkin M. Genealogy of neutral genes in two partially isolated populations. Theor Popul Biol. 1990;38: 331–350. 2293402 10.1016/0040-5809(90)90018-q

[39] Brand CL, Kingan SB, Wu L, Garrigan D. A selective sweep across species boundaries in *Drosophila* . Mol Biol Evol. 2013;30: 2177–2186. 10.1093/molbev/mst123 23827876 PMC3748358

[40] Kirkness EF, Grindberg RV, Yee-Greenbaum J, Marshall CR, Scherer SW, et al Sequencing of isolated sperm cells for direct haplotyping of a human genome. Genome Res. 2013;23: 826–832. 10.1101/gr.144600.112 23282328 PMC3638138

[41] Langley CH, Crepeau M, Cardeno C, Corbett-Detig R, Stevens K. Circumventing heterozygosity: sequencing the amplified genome of a single haploid *Drosophila melanogaster* embryo. Genetics. 2011;188: 239–246. 10.1534/genetics.111.127530 21441209 PMC3122310

