# Supplementary material for: A New Method to Scan Genomes for Introgression in a Secondary Contact Model
Source: PLoS One. 2015 Apr 14;10(4):e0118621. doi: 10.1371/journal.pone.0118621 (PMC4396994; doi:10.1371/journal.pone.0118621)
Supplement: S1 Table — (DOCX) [file pone.0118621.s011.docx]

**Supplementary Table 1**  **The sampled strains from two populations of *Drosophila melanogaster* used to contrast the performance of *F*_ST_ and *G*_min_, including short read archive accession numbers.**

| *population* | *stock* | *accession* |
| --- | --- | --- |
| France | FR14 | SRR189088 |
| France | FR151 | SRR189089 |
| France | FR207 | SRR189091 |
| France | FR217 | SRR189092 |
| France | FR229 | SRR189093 |
| France | FR310 | SRR189094 |
| France | FR361 | SRR189095 |
| Rwanda | RG10 | SRR189374 |
| Rwanda | RG11N | SRR189375 |
| Rwanda | RG15 | SRR189377 |
| Rwanda | RG15 | SRR189392 |
| Rwanda | RG21N | SRR189382 |
| Rwanda | RG32N | SRR189388 |
| Rwanda | RG34 | SRR189390 |
| Rwanda | RG35 | SRR189391 |
| Rwanda | RG35 | SRR189378 |
| Rwanda | RG36 | SRR189393 |
| Rwanda | RG38N | SRR189395 |
