# Supplementary material for: A New Method to Scan Genomes for Introgression in a Secondary Contact Model
Source: PLoS One. 2015 Apr 14;10(4):e0118621. doi: 10.1371/journal.pone.0118621 (PMC4396994; doi:10.1371/journal.pone.0118621)
Supplement: S3 Table — (DOCX) [file pone.0118621.s013.docx]

**Supplementary Table 3**  **Analysis of variance of specificity of the *F*_ST_ and *G*_min_ measures at varying quantile thresholds under a model including gene flow. Column values are the percent variance in each measure described by each parameter or interaction of parameters, filtered to include only those with an effect greater than 1%.**

|  | 5% *quantile* | |  | 1% *quantile* | |  | 0.1% *quantile* | |
| --- | --- | --- | --- | --- | --- | --- | --- | --- |
| *parameters* | *G*_min_ | *F*_ST_ |  | *G*_min_ | *F*_ST_ |  | *G*_min_ | *F*_ST_ |
| *τ*_D_ | 0.4 | 1.5 |  | 1.1 | 5.2 |  | 1.2 | 10.8 |
| *θ* | 0.7 | 0.1 |  | 0.8 | 0.6 |  | 1.1 | 5.2 |
| *ρ* | 1.2 | 5.8 |  | 0.7 | 7.7 |  | 0.2 | 0.8 |
| *n*_2_ | 14.9 | 13.3 |  | 11.6 | 9.7 |  | 10.3 | 1.9 |
| *λ* | 47.1 | 54.1 |  | 48.6 | 43.8 |  | 46.6 | 32.8 |
| *τ*_M_ | 6.4 | 0.9 |  | 9.0 | 2.5 |  | 13.2 | 6.9 |
| *τ*_D_ × *λ* | 0.2 | 0.9 |  | 0.1 | 2.4 |  | 0.1 | 2.2 |
| *ρ* × *P_M_* | 1.7 | 2.4 |  | 1.1 | 3.3 |  | 0.8 | 1.2 |
| *n*_2_ × *λ* | 2.7 | 7.3 |  | 1.2 | 2.5 |  | 0.3 | 0.0 |
| *τ*_D_ × *τ*_M_ | 0.3 | 0.3 |  | 0.7 | 0.8 |  | 0.3 | 1.5 |
| *θ* × *τ*_M_ | 0.5 | 0.0 |  | 0.5 | 0.3 |  | 0.5 | 1.6 |
| *ρ* × *τ*_M_ | 2.3 | 0.3 |  | 2.2 | 0.3 |  | 2.1 | 0.9 |
| *n2* × *τ*_M_ | 1.6 | 0.2 |  | 0.8 | 0.0 |  | 0.8 | 0.0 |
| *n*_2_ × *λ* × *τ*_M_ | 0.2 | 0.0 |  | 0.6 | 0.1 |  | 1.0 | 0.1 |
| coalescent | 16.0 | 10.1 |  | 18.8 | 17.6 |  | 19.6 | 30.3 |
