# Supplementary material for: A New Method to Scan Genomes for Introgression in a Secondary Contact Model
Source: PLoS One. 2015 Apr 14;10(4):e0118621. doi: 10.1371/journal.pone.0118621 (PMC4396994; doi:10.1371/journal.pone.0118621)
Supplement: S4 Table — (DOCX) [file pone.0118621.s014.docx]

**Supplementary Table 4**  **Sensitivity, specificity, and variance of both the *F*_ST_ and *G*_min_ statistics for varying values of the probability of migration (*λ*).**

| *migration probability* (λ) | *sensitivity* | |  | *specificity* | |  | *variance* | |
| --- | --- | --- | --- | --- | --- | --- | --- | --- |
|  | *G*_min_ | *F*_ST_ |  | *G*_min_ | *F*_ST_ |  | *G*_min_ | *F*_ST_ |
| 0.001 | 0.114 | 0.060 |  | 0.221 | 0.059 |  | 0.0230 | 0.0002 |
| 0.005 | 0.111 | 0.060 |  | 0.293 | 0.109 |  | 0.0232 | 0.0002 |
| 0.010 | 0.107 | 0.059 |  | 0.357 | 0.164 |  | 0.0234 | 0.0002 |
| 0.050 | 0.088 | 0.055 |  | 0.610 | 0.448 |  | 0.0246 | 0.0002 |
| 0.100 | 0.076 | 0.051 |  | 0.743 | 0.614 |  | 0.0255 | 0.0002 |
