# Supplementary material for: A New Method to Scan Genomes for Introgression in a Secondary Contact Model
Source: PLoS One. 2015 Apr 14;10(4):e0118621. doi: 10.1371/journal.pone.0118621 (PMC4396994; doi:10.1371/journal.pone.0118621)
Supplement: S1 Text — (DOCX) [file pone.0118621.s015.docx]

# A new method to scan genomes for introgression in a secondary contact model-- Supplementary Material

## In this supplement, we derive a basic statistical description of the minimum coalescent time between samples taken from populations with no post-divergence gene flow. We then relate these coalescent time results to the minimum number of nucleotide differences between populations ($\min\left(d_{XY}\right)$, the numerator of *G_min_*) and to the behavior of the overall *G_min_* statistic under these (null-model) conditions.

## *Model*

We assume a single panmictic population of size $N$ diploid individuals, split into two daughter populations (of size $N_{1}$ and $N_{2}$, respectively) $\tau_{D}$ generations in the past with no post-divergence migration. In the present time, we have sampled $n_{1}$ individuals from population 1 and $n_{2}$ individuals from population 2. Let $M$ be the number of generations before the present until the first coalescence between samples from different populations. Conditioning on $i$ and $j$ as the number of ancestral lineages remaining in each population at the time of divergence $\tau_{D}$, we can write the total time until the first between-population coalescence as

$$\begin{matrix} M=\text{ minimum between-population } \\ \text{ coalescence time in the sample} \end{matrix}$$

$=\tau_{D}+\sum_{i=1}^{n_{1}} \sum_{j=1}^{n_{2}} p_{n_{1},i}\left(\tau_{D},N_{1}\right)p_{n_{2},j}\left(\tau_{D},N_{2}\right)A_{i,j}.$ (S1)

Here, $A_{i,j}$ is the time (looking backwards from $\tau_{D}$) until the first coalescent event in the pre-divergence population involving one of the $i$ remaining lineages from (present-day) population 1 and one of the $j$ remaining lineages from population 2, and $p_{n_{k},m}\left(\tau_{D},N_{k}\right)$ is the probability that a sample of size $n_{k}$ taken from population $k$ has exactly $m$ ancestors at time $\tau_{D}$ generations before the time of sampling. We then have

$\text{E}\left[M\right]=\tau_{D}+\sum_{i=1}^{n_{1}} \sum_{j=1}^{n_{2}} p_{n_{1},i}\left(\tau_{D},N_{1}\right)p_{n_{2},j}\left(\tau_{D},N_{2}\right)\text{E}\left[A_{i,j}\right]$ (S2a)

and

$\text{Var}\left[M\right]=\sum_{i=1}^{n_{1}} \sum_{j=1}^{n_{2}} p_{n_{1},i}\left(\tau_{D},N_{1}\right)p_{n_{2},j}\left(\tau_{D},N_{2}\right)\text{Var}\left[A_{i,j}\right]$ (S2b)

as the mean and variance of the minimum between-population coalescence time under a null model of complete divergence. Expressions for $p_{n_{k},m}\left(\tau_{D},N_{k}\right)$ are well known [1]; we now turn to the derivation of the expected mean and variance of the time $A_{i,j}$, which are less well understood.

*Coalescent analysis of* $A_{i,j}$

In order to derive the mean and variance of $A_{i,j}$, the minimum between-population coalescence time in the ancestral population, we condition on the number of coalescent events that have occurred in the ancestral population prior to the first coalescent event joining lineages from both subsamples. It is important to note here that we are only considering the genealogy in the ancestral (pre-divergence) merged population, as the $p_{n_{k},m}\left(\tau_{D},N_{k}\right)$ probabilities account for the genealogical history in the daughter populations post-divergence. Starting, then, from the time of the population split $\tau_{D}$ and looking backwards, let $T_{k}$be the time of the first between-population coalescent event given $k$ prior ancestral coalescent events and $P_{k}$ the probability of $k$ ancestral coalescent events before the first between-population coalescent event. This yields

$\text{E}\left[A_{i,j}\right]=\sum_{k=0}^{i+j-2} P_{k}\text{E}\left[T_{k}\right]$ (S3)

and

$\text{Var}\left[A_{i,j}\right]=\sum_{k=0}^{i+j-2} P_{k}\text{Var}\left[T_{k}\right]+\sum_{k=0}^{i+j-2} P_{k}\text{E}\left[T_{k}\right]^{2}-\left(\sum_{k=0}^{i+j-2} P_{k}\text{E}\left[T_{k}\right]\right)^{2}$ (S4)

as expressions the mean and variance of the ancestral coalescent time.

From standard coalescent theory, we can readily write down $\text{E}\left[T_{k}\right]$, the expected time until a total of $k+1$ coalescent events ($k$ within-sample and one between-samples) have occurred in the ancestral merged population,

$\text{E}\left[T_{k}\right]=\frac{2\left(k+1\right)}{\left(i+j\right)\left[i+j-\left(k+1\right)\right]}$ (S5)

(where time is in units of $2N$generations), as well as the variance in that total time, given $k$:

$\text{Var}\left[T_{k}\right]=\sum_{l=0}^{k} \left(\frac{2}{\left(i+j-l\right)\left[i+j-\left(l+1\right)\right]}\right)^{2}.$ (S6)

To obtain $P_{k}$, begin with $P_{0}$, the probability that the first coalescent is between samples, and $P_{1}$, the probability that the first coalescent event is within a sample, immediately followed by the first between-sample event. These quantities can be written as

$P_{0}=\frac{2ij}{\left(i+j\right)\left[i+j-1\right]}$ (S7)

and

$$P_{1}=\left[\frac{i\left(i-1\right)}{\left(i+j\right)\left(i+j-1\right)}\right]\left[\frac{2\left(i-1\right)j}{\left(i+j-1\right)\left(i+j-2\right)}\right]$$

$+\left[\frac{j\left(j-1\right)}{\left(i+j\right)\left(i+j-1\right)}\right]\left[\frac{2i\left(j-1\right)}{\left(i+j-1\right)\left(i+j-2\right)}\right]$ (S8)

$P_{1}$ was obtained by considering the two possible paths by which a single prior coalescence could have happened. A full general formula for $P_{k}$ is obtained by extending this logic and summing over the possible paths by which $k$prior coalescent events could have occurred,

(S9)

The above expressions yield an expression for the expectation of the minimum between-population coalescence time from the time of divergence

(S10)

and $\text{Var}\left[A_{i,j}\right]$ can now also be obtained using equations S4, S6, and S9. The mean and variance of the overall expected minimum time to between-population coalescence under the null-model assumptions of population divergence and complete isolation at time $\tau_{D}$ follow immediately from these results applied to equations S2a and S2b.

Relatively simple solutions for both mean and variance of $A_{i,j}$ are also available for the special case in which only one ancestral lineage is present in at least one population at the time of divergence ($j=1$):

$P_{k}=\frac{2\left(i-k\right)}{i\left(i+1\right)}$ (S11)

$\text{E}\left[A_{i,1}\right]=\frac{2}{i+1}$ (S12)

(S13)

*Relation to* G_min_

The minimum between-population coalescence time is closely related to the numerator of the descriptive statistic *G_min_* ,$\min\left(d_{XY}\right)$. We cannot directly use the results above to develop a formal statistical description of *G_min_*, however, for two reasons. First, because *G_min_* is a ratio of two (related) random variables, a rigorous statistical treatment of *G_min_* requires derivation of quantities such as the covariance between numerator and denominator, currently unknown. Additionally, the relationship between minimum coalescence time and minimum number of mutations is complicated by the fact that samples with the minimum number of differences do not necessarily occur along the shortest genealogical branches of a tree. There is an extra level of sampling from the extremes of a stochastic process (mutation) that we have not accounted for in our treatment of the extreme value of the genealogical process. We expect this problem, leading to systematic biases in the expected mean and variance of the number of nucleotide differences under our null model, to be particularly acute for low mutation rates ($\theta$).

Despite these difficulties, an application of a Poisson model of mutation to the minimum coalescent times derived above can provide some basic insights into the properties of the *G_min_* statistic. By assuming a Poisson model of mutation (with rate $\mu=\frac{\theta}{4N}$, where $N=N_{1}=N_{2}$ for simplicity) along lineages, we can write down the following approximate expressions for the mean and variance of the numerator of *G_min_*, $\min\left(d_{XY}\right)$, in terms of the mean and variance of the minimum coalescent time and the other parameters:

$$\text{E}\left[\text{min}\left(d_{XY}\right)\right]=\text{E}\left[2\mu\left(\tau_{D}+\sum_{i=1}^{n_{1}} \sum_{j=1}^{n_{2}} p_{n_{1},i}\left(\tau_{D},N_{1}\right)p_{n_{2},j}\left(\tau_{D},N_{2}\right)A_{i,j}\right)\right]$$

$=2\mu\left(\tau_{D}+\sum_{i=1}^{n_{1}} \sum_{j=1}^{n_{2}} p_{n_{1},i}\left(\tau_{D},N_{1}\right)p_{n_{2},j}\left(\tau_{D},N_{2}\right)\text{E}\left[A_{i,j}\right]\right)$ (S14)

$$\text{Var}\left[\text{min}\left(d_{XY}\right)\right]=\text{E}\left[\text{min}\left(d_{XY}\right)\right]+\text{Var}\left[2\mu\left(\tau_{D}+\sum_{i=1}^{n_{1}} \sum_{j=1}^{n_{2}} p_{n_{1},i}\left(\tau_{D},N_{1}\right)p_{n_{2},j}\left(\tau_{D},N_{2}\right)A_{i,j}\right)\right]$$

$=\text{E}\left[\text{min}\left(d_{XY}\right)\right]+4\mu^{2}\sum_{i=1}^{n_{1}} \sum_{j=1}^{n_{2}} p_{n_{1},i}\left(\tau_{D},N_{1}\right)p_{n_{2},j}\left(\tau_{D},N_{2}\right)\text{Var}\left[A_{i,j}\right]$ (S15)

We note that the resulting approximate E[$\min\left(d_{XY}\right)$] (obtained from substitution of our expressions for $\text{E}\left[A_{i,j}\right]$ and $\text{Var}\left[A_{i,j}\right]$) will be of order $\theta$ while Var[$\min\left(d_{XY}\right)$] has both a $\theta$and $\theta^{2}$term. This is in accordance with the expectation and variance of the denominator of the *G_min_* , ($\bar{d}_{XY}$) as derived by Takahata [2], who gave the expectation as

$$\text{E}\left[\bar{d}_{XY}\right]=\theta\left(\tau_{D}+1\right)$$

The variance of the *G_min_* denominator as derived by Takahata [2] is more complicated, but has accessible approximations when $\tau_{D}$ is very large (>>1),

$$\text{Var}\left[\bar{d}_{XY}\right]=\theta\left(\tau_{D}+\theta\right),$$

or very small (<<1),

$$\text{Var}\left[\bar{d}_{XY}\right]=\frac{1}{3}\theta+\frac{2}{9}\theta^{2}.$$

These results imply that the behavior of *G*_min_ should be relatively insensitive to values of $\theta$, consistent with our numerical simulations. The value of the *G*_min_ ratio is, through $\min\left(d_{XY}\right)$, predominantly influenced by population factors affecting the number of lineages present in the samples at the time of divergence. We note that, for example, increasingly remote divergence times will result in small numbers of ancestral lineages, increasing the expected value of *G*_min_. In the limit of one lineage per sample, $\text{E}\left[A_{1,1}\right]$ is one, and the numerator and denominator of *G*_min_ are equal in expectation. For the slightly less extreme case where there are two lineages remaining in one sample, $\text{E}\left[A_{1,2}\right]=\frac{2}{3}$, yielding a *G*_min_ expectation close to one for $\tau_{D}$greater than one. A bottleneck in one of the descendent populations will likely lead to a small number of lineages present at divergence, and a *G*_min_ close to one is again expected. Thus, we posit that for a range of realistic population scenarios, the null expectation for *G_min_* should be extreme (approaching 1), giving us considerable potential to detect introgression events that constitute a departure from that null.

## Supplement References

1. Tavaré S. Line-of-descent and genealogical processes, and their applications in population genetics models. Theor Popul Biol. 1984;26: 119-164.

2. Takahata N, Nei M. Gene genealogy and variance of interpopulational nucleotide differences. Genetics. 1985;110: 325-334.
